# Puerarin Protects against Cardiac Fibrosis Associated with the Inhibition of TGF-*β*1/Smad2-Mediated Endothelial-to-Mesenchymal Transition

**DOI:** 10.1155/2017/2647129

**Published:** 2017-05-30

**Authors:** Ya-Ge Jin, Yuan Yuan, Qing-Qing Wu, Ning Zhang, Di Fan, Yan Che, Zhao-Peng Wang, Yang Xiao, Sha-Sha Wang, Qi-Zhu Tang

**Affiliations:** ^1^Department of Cardiology, Renmin Hospital of Wuhan University, Wuhan, China; ^2^Cardiovascular Research Institute of Wuhan University, Wuhan, China; ^3^Hubei Key Laboratory of Cardiology, Wuhan, China

## Abstract

**Background:**

Puerarin is a kind of flavonoids and is extracted from Chinese herb Kudzu root. Puerarin is widely used as an adjuvant therapy in Chinese clinics. But little is known about its effects on regulating cardiac fibrosis.

**Methods:**

Mice were subjected to transverse aorta constriction (TAC) for 8 weeks; meanwhile puerarin was given 1 week after TAC. Cardiac fibrosis was assessed by pathological staining. The mRNA and protein changes of CD31 and vimentin in both animal and human umbilical vein endothelial cells (HUVECs) models were detected. Immunofluorescence colocalization of CD31 and vimentin and scratch test were carried out to examine TGF-*β*1-induced changes in HUVECs. The agonist and antagonist of peroxisome proliferator-activated receptor-*γ* (PPAR-*γ*) were used to explore the underlying mechanism.

**Results:**

Puerarin mitigated TAC-induced cardiac fibrosis, accompanied with suppressed endothelial-to-mesenchymal transition (EndMT). The consistent results were achieved in HUVECs model. TGF-*β*1/Smad2 signaling pathway was blunted and PPAR-*γ* expression was upregulated in puerarin-treated mice and HUVECs. Pioglitazone could reproduce the protective effect in HUVECs, while GW9662 reversed this effect imposed by puerarin.

**Conclusion:**

Puerarin protected against TAC-induced cardiac fibrosis, and this protective effect may be attributed to the upregulation of PPAR-*γ* and the inhibition of TGF-*β*1/Smad2-mediated EndMT.

## 1. Introduction

Cardiac fibrosis is a final common pathway of a wide spectrum of heart diseases, including myocardial infarction [1], ischemia-reperfusion injury [2], hypertrophic cardiomyopathy [3], and diabetic cardiomyopathy [4]. Cardiac fibrosis is characterized by uncontrolled and excessive accumulation of extracellular matrix (ECM) and collagen, which increase the ventricular stiffness, deteriorate the diastolic function, and eventually lead to heart failure. Myofibroblasts, expressing *α*-smooth muscle actin (*α*-SMA) and exhibiting huge capability of producing ECM and collagen (types I and III), as well as inhibiting the activity of matrix metalloproteinases (MMPs), are known as the main effector cells responsible for cardiac fibrosis. Myofibroblasts can originate from quiescent fibroblasts in situ, which can be activated and transdifferentiate into myofibroblasts rapidly after heart injuries [5]; bone marrow-derived circulating fibrocytes, attracted by different kinds of cytokines and chemokines, migrate and accumulate in myocardium, representing another important source of myofibroblasts [6]; recent studies have reported another source of myofibroblasts: to cope with different kinds of stress, epithelial/endothelial cells lose their intrinsic characteristics and acquire mesenchymal cell characteristics, a process known as epithelial/endothelial-to-mesenchymal transition (EMT/EndMT) [7, 8]. First proposed by Karasek [9], EMT/EndMT has been continuously shown to play an important role in fibrosis and should be given enough attention.

Puerarin (7,4′-dihydroxy-8-*β*-D-glucosylisoflavone, C_21_H_20_O_9_) (Pue) [10] is a kind of flavonoids, which is extracted from Chinese herb Kudzu root and widely used in Chinese clinics as an adjuvant therapy for the treatment of angina pectoris, diabetes [11], and ischemic cerebrovascular diseases [12]. Our previous [13] and others' studies [14] have demonstrated that puerarin attenuated pressure or angiotensin II-induced cardiac hypertrophy in mice, and puerarin could also inhibit inflammation and apoptosis in LPS-stimulated cardiomyocytes [15]. However, there is a lack of data on puerarin's effects on cardiac fibrosis. Our present study is aimed at elucidating the protective effect of puerarin on cardiac fibrosis induced by transverse aorta constriction (TAC).

## 2. Materials and Methods

### 2.1. Chemicals and Reagents

Puerarin (98% purity as detected by high-performance liquid chromatography analysis) was acquired from Shanghai Winherb Medical S&T Development Co. Ltd. (Shanghai, China). TGF-*β*1 was purchased from PEPROTECH (100-21C). GW9662 was purchased from Sigma (M6191) and pioglitazone was also purchased from Sigma (CDS021593).

### 2.2. Animals

All animal procedures used in this study were approved by the Animal Care and Use Committee of Renmin Hospital of Wuhan University (protocol number: 00013274) and conformed to the Care and Use of Laboratory Animals published by the US National Institutes of Health (NIH Publication number 85-23, revised 1996). 8-week-old, male C57/BL6 mice weighing 23.5–27.5 g were purchased from the Institute of Laboratory Animal Science, CAMS & PUMC (Beijing, China) and kept in specific pathogen free animal lab with 12 h light-dark cycle and constant temperature and humidity. After 1 week of adaptation, the mice were randomly assigned to four groups, as sham + vehicle, sham + pue, TAC + vehicle, and TAC + pue, among which vehicle or puerarin were given 1 week after TAC. The dose (65 mg/kg body weight/day) and administration route (premixed in daily feed) of puerarin were determined by referring to our previous study [13]. All the mice had free access to drinking water and feed. Food consumption was monitored once a week and no difference was found between the four groups. The chosen dose of puerarin had no adverse effects on their growth or the food and water consumption. 8 weeks after sham or TAC, the mice were sacrificed and their hearts were taken for further studies.

### 2.3. Cell Culture

Human umbilical vein endothelial cell (HUVEC) line 12 was purchased from YRGene (NC006) and cultured in Gibco RPMI1640 medium supplemented with 10% fetal bovine serum (FBS), endothelial cell growth supplement (ECGS, ScienCell, 1052, 10 *μ*l/ml), and heparin (0.1 mg/ml) in a humidified 5% CO_2_ incubator at 37°C. Cells between the fourth and sixth passages growing to 70–80% confluence were used.

After 12 hours of starvation for synchronization, HUVECs were preincubated with different concentrations of puerarin (10 *μ*M, 25 *μ*M, and 50 *μ*M) or pioglitazone (20 *μ*M) in the presence or absence of GW9662 (10 *μ*M) for 30 minutes and then incubated with recombinant human TGF-*β*1 (10 ng/ml) or phosphate buffer saline (PBS) for 48 hours. The concentrations of puerarin were chosen by referring to the previous studies [14, 15]. The whole cell lysates and RNA were extracted for further study. Puerarin, pioglitazone, and GW9662 were predissolved in dimethyl sulfoxide (Sigma, D2650) and the chosen concentrations showed no harmful effects.

### 2.4. Western Blotting

Protein samples from mice hearts or HUVECs lysates were assessed using BCA-kit (23227, Thermo Fisher Scientific, Waltham, MA, USA) and normalized to the same concentration before all western blotting (WB) experiments. 50 *μ*g of protein samples were separated by sodium dodecyl sulphate polyacrylamide gel electrophoresis and then transferred to Immobilon-FL transfer membrane (IPFL00010, Millipore, Billerica, MA, USA). The membranes were blocked with 5% milk in Tris-buffered saline Tween-20 (TBST) for 3 hours and then incubated with indicated primary antibodies overnight at 4°C. Antibodies against the following proteins were purchased from Cell Signaling Technology (Danvers, MA, USA): Smad2 (#3103s), glyceraldehyde-3-phosphate dehydrogenase (GAPDH) (#2118), and phosphor-Smad2^Ser465/467^ (#3101). Antibodies against the following proteins were purchased from Abcam (Cambridge, MA, USA): alpha smooth muscle actin (*α*-SMA) (#ab7817), TGF-beta 1 (#ab66043), and CD31 (#ab24590). Antibodies against the following proteins were purchased from Santa Cruz Biotechnology (Santa Cruz, CA, USA): peroxisome proliferator-activated receptor-*γ* (PPAR-*γ*) (#sc-7196), Smad4 (#sc-7966), and vimentin (#sc-5565). The blots were scanned by a two-color infrared imaging system (Odyssey, LI-COR, Lincoln, NE, USA). Specific protein expression levels were normalized to GAPDH protein for total cell lysates.

### 2.5. Quantitative Real-Time Reverse Transcription-Polymerase Chain Reaction

RNA was extracted from HUVECs using TRIzol (Invitrogen, 15596-026) and reversely transcribed into cDNA for real-time polymerase chain reaction (RT-PCR) analysis using oligo (DT) primers and the Transcriptor First-Strand cDNA Synthesis Kit (04896866001, Roche, Basel, Switzerland). cDNA was synthesized from 2 *μ*g of total RNA. The PCR amplifications were quantified using a LightCycler 480 SYBR Green I Master Mix (Roche, 04707516001) and the results were normalized against GAPDH gene expression. The primers used were showed in Table 1.

### 2.6. Scratch Adhesion Test

HUVECs were seeded in six-well plates until they formed a confluent monolayer. After 12 hours of starvation, HUVECs were pretreated with different concentrations of puerarin (10 *μ*M, 25 *μ*M, and 50 *μ*M) for 30 minutes and then treated with PBS or TGF-*β*1 (10 ng/ml) for 48 hours. Scratches were made across the HUVECs monolayer with a 200 *μ*l sterilized micropipette tip and the plates were rinsed twice with sterilized PBS. Meanwhile to arrest cell proliferation the medium was replaced by Gibco RPMI1640 medium supplemented with no FBS. Photographs were taken at 0, 6, 12, and 24 hours after scarification using inverted microscope (IX51, Olympus, Tokyo, Japan) to observe the migration of HUVECs.

### 2.7. Histological Analysis and Immunohistochemistry

Mice hearts were taken and fixed in 10% formalin after being arrested in diastolic phase with 10% KCl and then embedded in paraffin. These paraffin-embedded samples were sectioned into 4-5 *μ*m thick slices perpendicular to the apex to make sure both ventricles fully manifested. Picrosirius red (PSR) staining was performed to evaluate the fibrosis level. Images were captured at 200x magnification from 5 fields per case. To detect the expression and location of *α*-SMA, the paraffin-embedded heart sections were blocked in 3% H_2_O_2_, stained with primary antibody against *α*-SMA (1 : 50) at 4°C overnight, and then incubated with peroxidase-coupled secondary antibody and DAB as a substrate. Images were captured at 400x magnification from 5 fields per case.

### 2.8. Immunofluorescence

The colocalization of CD31 and vimentin was tested with immunofluorescence. The frozen sections of mice hearts were put at room temperature for at least 1 hour until they dried out. After soaking into 0.3% H_2_O_2_ (diluted in TBS 1x) for 30 minutes, the sections were rinsed with TBS (1x) for 5 minutes and then blocked with 10% goat serum (Gibco, 16210-064) for 10 minutes before incubation with primary antibodies against CD31 (Abcam, ab24590) and vimentin (Santa Cruz, sc-5565) at 37°C for 2 hours. After that, the sections were rinsed with TBS for 15 minutes and incubated with two different IRDye® 800CW-conjugated secondary antibodies for 60 minutes at room temperature. After 15-min rinsing, the sections were counterstained by SlowFade Gold antifade reagent containing 4′-6-diamidino-2-phenylindole (DAPI, S36939, Invitrogen) and then observed under inverted microscope. For HUVECs immunofluorescence, there were some extra steps: before serum blocking, HUVECs which adhered to cell climbing pieces (12-545, Fisher Scientific, USA), needed to be fixed with 4% paraformaldehyde for 10 minutes and permeabilized with 0.2% Triton X-100 (0694, Amresco, Penn, USA) for 5 minutes at room temperature.

### 2.9. Statistical Analysis

Data were shown as mean ± SEM. The significance in difference between groups was tested by one-way analysis of variance (ANOVA) test followed by a post hoc Tukey test using SPSS 22.0 statistical software (SPSS, Chicago, USA). A two-tailed *p* < 0.05 was considered significant.

## 3. Results

### 3.1. Puerarin Attenuated TAC-Induced Cardiac Fibrosis in Mice

**Figure 1:**
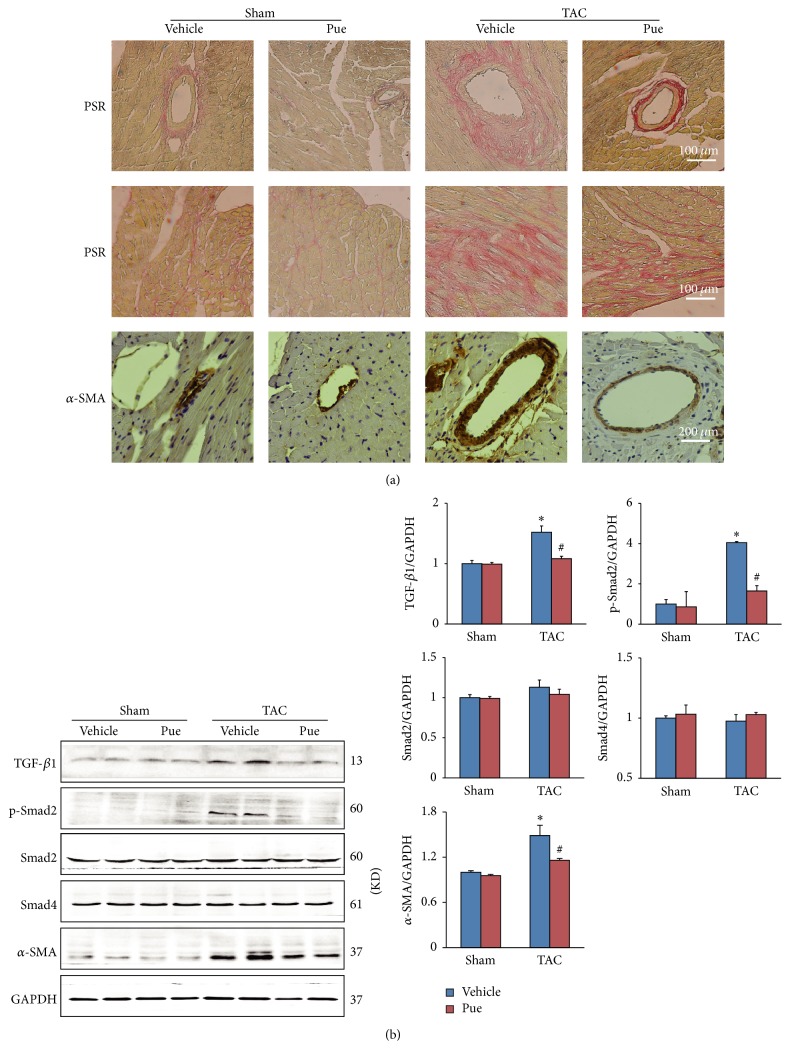
*Pressure overload-induced cardiac fibrosis was alleviated in puerarin-treated mice*. (a) Histological sections of the left ventricle in indicated groups were stained with PSR for fibrosis (upper and middle panel, scale bars: 100 *μ*m). *α*-SMA was detected with immunohistochemistry (lower panel, scale bars: 200 *μ*m). (b) TGF-*β*1/Smad2 signaling pathway and *α*-SMA protein in indicated groups were determined by WB, normalized to GAPDH (*n* = 6). ^**∗**^*p* < 0.05 versus sham + vehicle group; ^#^*p* < 0.05 versus TAC + vehicle group.

After 8 weeks of TAC, the mice hearts in TAC + vehicle group showed prominent fibrosis compared with sham groups as evidenced by PSR staining (Figure 1(a)). Puerarin administration significantly decreased the deposition of extracellular matrix and collagen in myocardium. It was noteworthy that the *α*-SMA positive staining, a special sign for myofibroblast, was much sparser in perivascular space in mice of TAC + Pue group compared with TAC + vehicle group (Figure 1(a)). The consistent result was seen in western blotting as *α*-SMA protein level was significantly downregulated (Figure 1(b)). And the blunted TGF-*β*1/Smad2 signaling proteins (Figure 1(b)) provided another evidence for puerarin's suppression effect on cardiac fibrosis. These hints indicated that puerarin attenuated TAC-induced cardiac fibrosis and this protective effect may have something to do with the vessels in the heart.

### 3.2. Puerarin's Protective Effect on Cardiac Fibrosis in Mice Was Involved with EndMT

**Figure 2:**
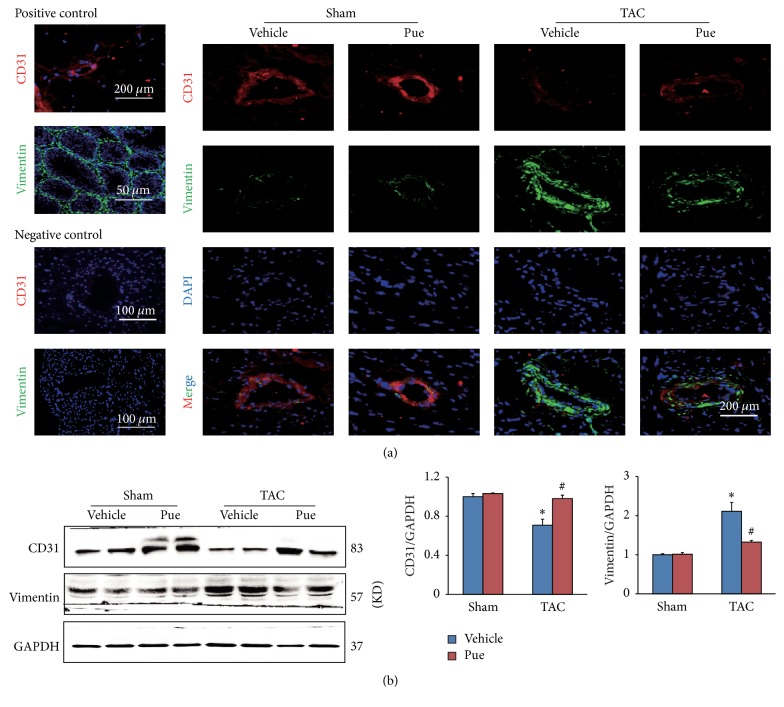
*Puerarin protected against cardiac fibrosis by inhibiting EndMT*. (a) Immunofluorescence colocalization of CD31 (red) and vimentin (green) was tested on frozen sections of mice hearts in indicated groups (scale bars: 200 *μ*m). The left panel was positive and negative controls. Positive control: CD31 (red) counterstained with DAPI (blue) in the muscle of mouse; vimentin (green) counterstained with DAPI (blue) in the testis of mouse. Negative control: heart tissue incubated with PBS instead of primary antibodies against CD31 or vimentin. (b) Protein expressions of CD31 and vimentin were determined by WB, normalized to GAPDH (*n* = 6). ^**∗**^*p* < 0.05 versus sham + vehicle group; ^#^*p* < 0.05 versus TAC + vehicle group.

Puerarin did inhibit TAC-induced cardiac fibrosis but how? Some researches [16, 17] found that EndMT provided an important source of fibroblasts and contributed to cardiac fibrosis in pathological conditions associated with pressure overload. We hypothesized that puerarin's protective effect may be associated with the inhibition of EndMT. Immunofluorescence on frozen heart sections was carried out to test this hypothesis. First of all, to test the sensitivity of CD31 and vimentin antibodies, we used mouse muscle tissue which is abundant in vessels and mouse testis tissue which is abundant in mesenchymal cells, as positive controls (Figure 2(a), left panel). To test the specificity of the two antibodies, we used mouse heart tissue incubated with PBS instead of primary antibodies against CD31 or vimentin, as negative controls (Figure 2(a), left panel). TAC induced a significant increase of mesenchymal cell marker vimentin (green) and a marked decrease of endothelial cell marker CD31 (red), as noted in Figure 2(a). These changes indicated that part of mesenchymal cells originating from endothelial cells contributed to TAC-induced cardiac fibrosis. However with puerarin administration, this trend was evidently redeemed, or in other words EndMT process was blocked, as shown by the downregulated vimentin and upregulated CD31 in TAC + Pue group. The consistent results were achieved in western blotting (Figure 2(b)). These results indicated that puerarin protected against TAC-induced cardiac fibrosis and this effect was involved with the suppression of EndMT.

### 3.3. Puerarin Inhibited EndMT in HUVECs Treated with TGF-*β*1

**Figure 3:**
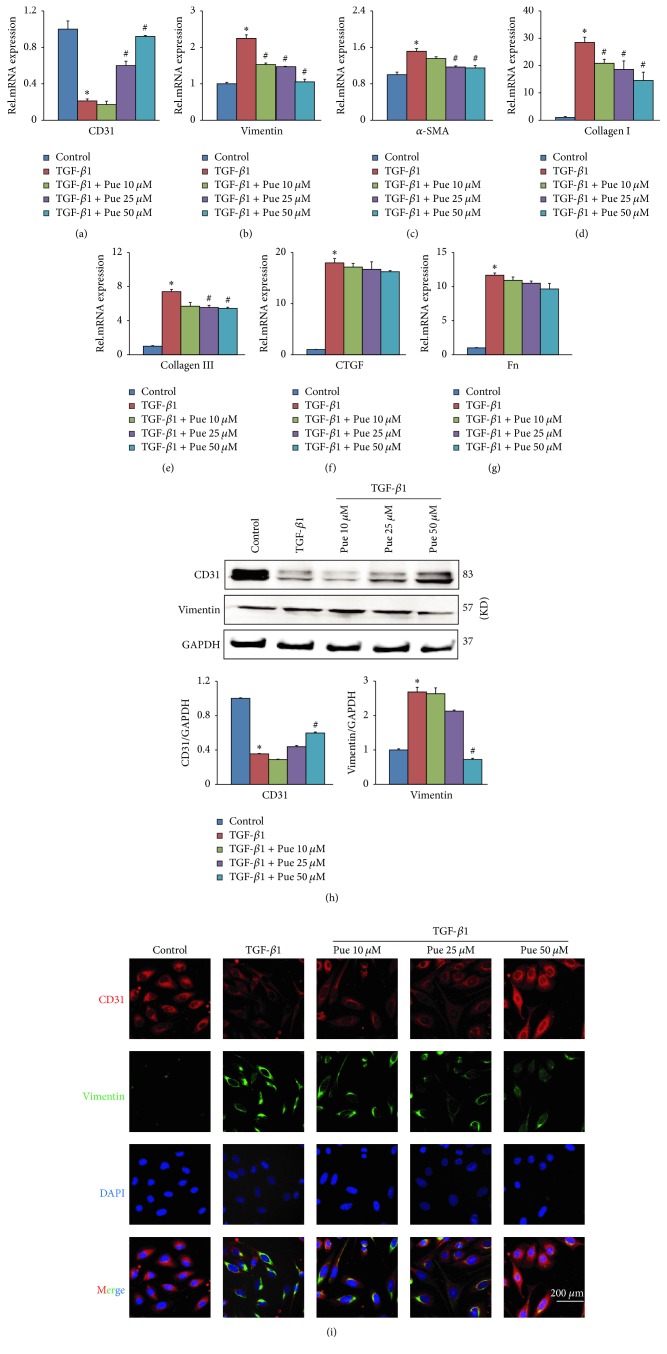
*Puerarin inhibited TGF-β1-induced EndMT in HUVECs*. (a–g) HUVECs were preincubated with different concentrations of puerarin (10, 25, and 50 *μ*M) for 30 min and then treated with TGF-*β*1 (10 ng/ml) for 48 h. mRNA levels of CD31, vimentin, *α*-SMA, collagen I, collagen III, CTGF, and Fn in indicated groups were tested by RT-PCR, normalized to GAPDH (*n* = 6). ^**∗**^*p* < 0.05 versus control group; ^#^*p* < 0.05 versus TGF-*β*1 group. (h) HUVECs were treated in the same way as mentioned above. The protein levels of CD31 and vimentin in cell lysates in indicated groups were tested by WB. ^**∗**^*p* < 0.05 versus control group; ^#^*p* < 0.05 versus TGF-*β*1 group. (i) HUVECs were treated in the same way as mentioned above. Immunofluorescence colocalization of CD31 (red) and vimentin (green) counterstained with DAPI (blue) was carried out in indicated groups (scale bars: 200 *μ*m).

To further concrete this theory, we established an in vitro model using HUVECs treated with TGF-*β*1 (10 ng/ml) for continuous 48 hours, as TGF-*β*1 was proven to effectively trigger the EndMT process [16]. HUVECs were pretreated with gradient concentrations of puerarin (10, 25, and 50 *μ*M) for 30 minutes and then incubated with TGF-*β*1 for 48 hours. The control group was treated with PBS instead of TGF-*β*1 for the same time. EndMT model was successfully built as shown in RT-PCR and western blotting results (Figures 3(a), 3(b), and 3(h)): there was a significant decrease of CD31 in both mRNA and protein level accompanied by a significant increase of vimentin in both mRNA and protein level. In addition to CD31 and vimentin, fibrosis activation was also obvious in TGF-*β*1 group as noted by the elevated mRNA level of *α*-SMA, collagen I, collagen III, CTGF, and Fn (Figures 3(c)–3(g)). However, puerarin pretreatment could markedly buffer the opposite changes of CD31 and vimentin (Figures 3(a), 3(b), and 3(h)) and also alleviated fibrosis activation (Figures 3(c), 3(d), and 3(e)), although the mRNA levels of CTGF and Fn showed no statistical differences as compared to TGF-*β*1 group. Another considerable result was that the suppression effect on EndMT and fibrosis was dose-dependent and the best result was achieved at 50 *μ*M. The consistent results could be seen in immunofluorescence colocalization result of CD31 (red) and vimentin (green) (Figure 3(i)).

### 3.4. Puerarin Inhibited TGF-*β*1-Induced HUVECs Migration Rate

**Figure 4:**
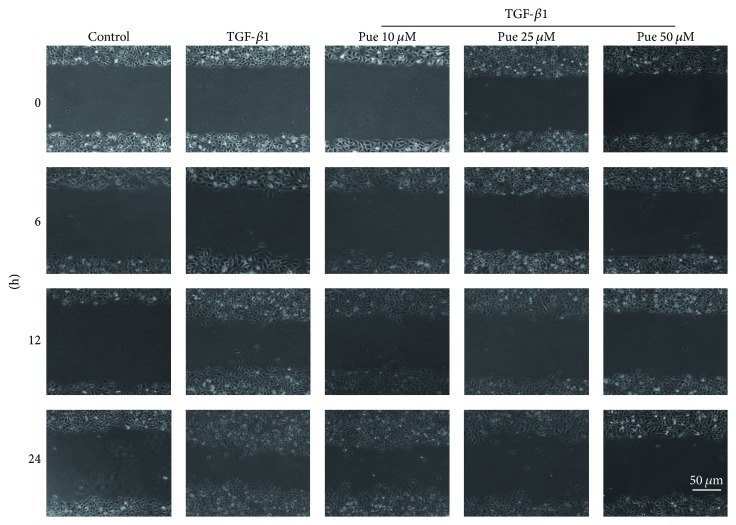
*Puerarin *inhibited* TGF-β1-induced HUVECs migration rate*. HUVECs were preincubated with different concentrations of puerarin (10, 25, and 50 *μ*M) for 30 min and then treated with TGF-*β*1 (10 ng/ml) for 48 h until a monolayer was formed. Scratches were made straightly across the dishes. After rinsing twice, the medium was replaced by RPMI1640 with no FBS. Photographs were taken at indicated times (scale bars: 50 *μ*m).

Except for phenotypic change, the alteration of cell biological behavior is another important characteristic of EndMT [18, 19]. We next performed scratch test to inspect puerarin's influence on TGF-*β*1-induced HUVECs' migration rate. Under inverted microscope (Figure 4), in addition to a spindle-like shape change, HUVECs in TGF-*β*1 group showed more cell movement along the originally neat scratch edges at 6 hours after scarification compared with control group and this movement peaked at the end of our observation phase which was 24 hours after scarification. This shape and behavior change indicated HUVECs' acquisition of mesenchymal cell's migration ability, which was another strong evidence of EndMT. However, puerarin could efficiently blunt HUVECs' changes triggered by TGF-*β*1, as noticed by the neater edges of scratch marks. Similarly, this effect was dose-dependent and at 50 *μ*M dose, puerarin exerted its best inhibition effect compared with the other two doses.

### 3.5. Puerarin's Inhibition on EndMT Was Associated with Inhibiting TGF-*β*1/Smad2 Pathway in HUVECs

**Figure 5:**
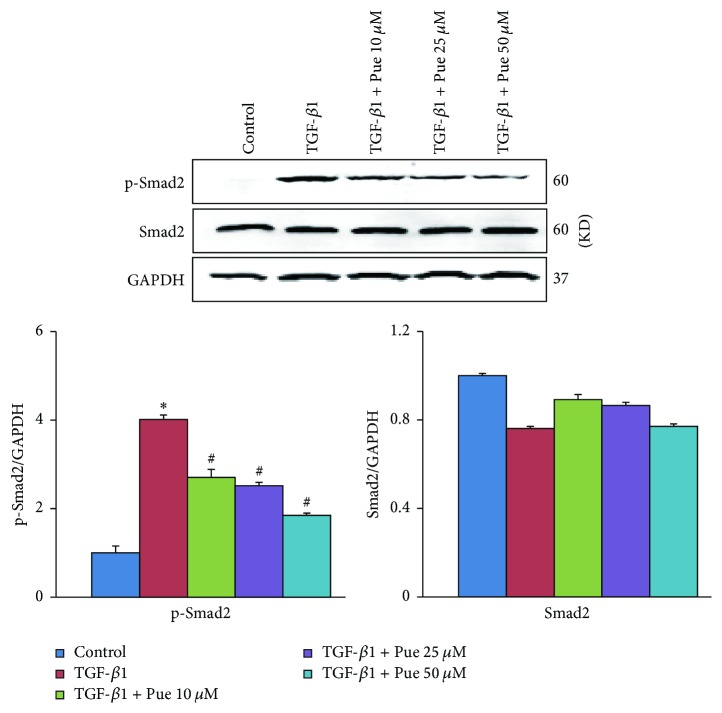
*Puerarin inhibited Smad2 phosphorylation in HUVECs. *HUVECs were preincubated with different concentrations of puerarin (10, 25, and 50 *μ*M) for 30 min and then treated with TGF-*β*1 (10 ng/ml) for 48 h. Protein levels of p-Smad2 and Smad2 in cell lysates in indicated groups were detected by WB, normalized to GAPDH (*n* = 6). ^*∗*^*p* < 0.05 versus control group; ^#^*p* < 0.05 versus TGF-*β*1 group.

Given the crucial role of canonical TGF-*β*1/Smads signaling pathway in the pathogenesis of numerous fibrotic diseases, we tested the downstream molecule of TGF-*β*1. As shown in Figure 5, TGF-*β*1-induced Smad2 phosphorylation activation was inhibited in a dose-dependent way, which was consistent with the mitigated EndMT and fibrosis after puerarin treatment (Figures 3 and 4).

### 3.6. Puerarin May Exert Beneficial Effect via PPAR-*γ* Upregulation

**Figure 6:**
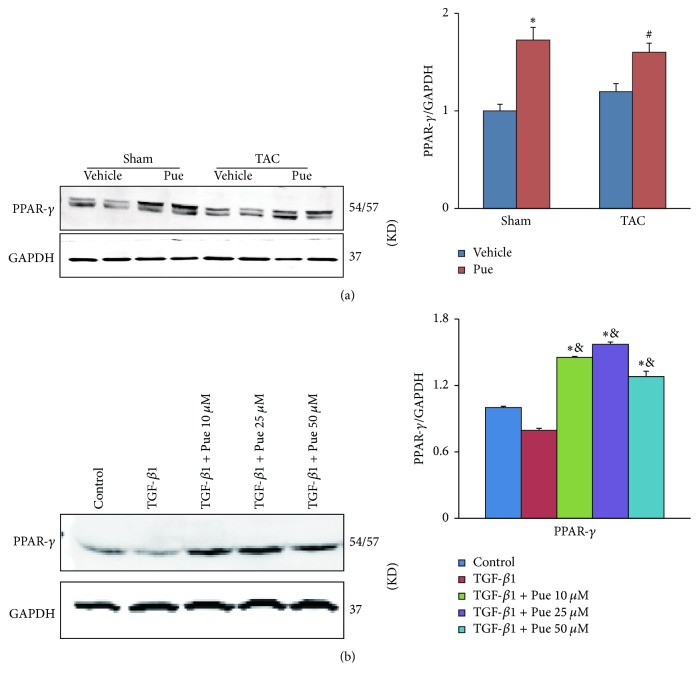
*PPAR-γ protein expression was upregulated by puerarin*. (a) PPAR-*γ* protein levels in mice hearts in indicated groups were detected by WB, normalized to GAPDH (*n* = 6). ^**∗**^*p* < 0.05 versus sham + vehicle group; ^#^*p* < 0.05 versus TAC + vehicle group. (b) HUVECs were preincubated with different concentrations of puerarin (10, 25, 50 *μ*M) for 30 min and then treated with TGF-*β*1 (10 ng/ml) for 48 h. PPAR-*γ* protein levels in cell lysates in indicated groups were detected by WB, normalized to GAPDH (*n* = 6). ^*∗*^*p* < 0.05 versus control group; ^&^*p* < 0.05 versus TGF-*β*1 group.

Surprisingly, we noticed that peroxisome proliferator-activated receptor-*γ* (PPAR-*γ*) protein level was upregulated in mice and HUVECs treated with puerarin (Figures 6(a) and 6(b)). PPAR-*γ* is well known for its role in negatively regulating fibrosis and EMT [20–22]. Did increased PPAR-*γ* have anything to do with puerarin's beneficial effect? If it did, what was the relationship between PPAR-*γ* and puerarin? These doubts drove us to do further study.

### 3.7. GW9662 Counteracted Puerarin's Suppression Effect on EndMT

**Figure 7:**
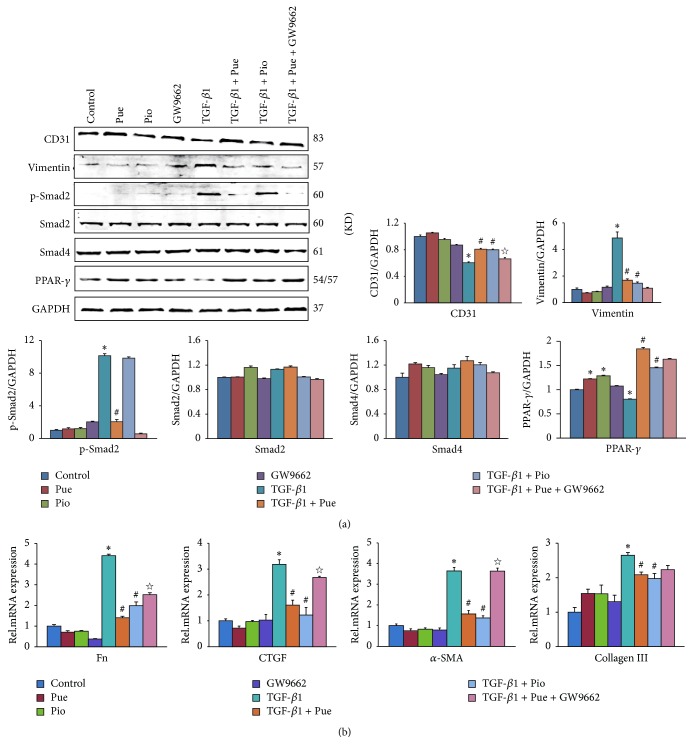
*GW9662 counteracted puerarin's suppression effect on EndMT. *(a) HUVECs were preincubated with puerarin (50 *μ*M) or pioglitazone (20 *μ*M) in the presence or absence of GW9662 (10 *μ*M) for 30 min and then treated with TGF-*β*1 (10 ng/ml) for 48 h. The protein levels of CD31, vimentin, p-Smad2, Smad2, Smad4, and PPAR-*γ* in cell lysates of indicated groups were detected by WB, normalized to GAPDH (*n* = 6). ^**∗**^*p* < 0.05 versus control group; ^#^*p* < 0.05 versus TGF-*β*1 group; ^☆^*p* < 0.05 versus TGF-*β*1 + Pue group. (b) HUVECs were treated in the same way as mentioned above. mRNA levels of CD31, vimentin, Fn, CTGF, *α*-SMA, and collagen III in indicated groups were tested by RT-PCR, normalized to GAPDH (*n* = 6). ^**∗**^*p* < 0.05 versus control group; ^#^*p* < 0.05 versus TGF-*β*1 group; ^☆^*p* < 0.05 versus TGF-*β*1 + Pue group.

To explore the relationship between puerarin and PPAR-*γ*, we used exogenous PPAR-*γ* agonist, pioglitazone (Pio), a drug used to treat type 2 diabetes mellitus, to pretreat HUVECs before the intervention of TGF-*β*1. As the western blotting and RT-PCR results showed (Figure 7), pioglitazone exerted the same suppression effect on TGF-*β*1-induced increase of vimentin, as well as other profibrotic genes, and decrease of CD31 to the extent of what puerarin did in TGF-*β*1 + Pue group. However with GW9662, a specific antagonist of PPAR-*γ*, the suppression effect imposed by puerarin was partially sabotaged: the CD31 protein and the profibrotic genes Fn, CTGF, and *α*-SMA between TGF-*β*1 + Pue and TGF-*β*1 + Pue + GW9662 group were statistically different. But the mRNA levels of Fn and CTGF were not as high as that in the TGF-*β*1 group.

## 4. Discussion 

In this study, we found that puerarin could inhibit pressure overload-induced cardiac fibrosis and this protective effect may be exerted by upregulation of PPAR-*γ* and suppressing TGF-*β*1/Smad2-mediated EndMT process.

Puerarin has been broadly applied in China due to its wide spectrum of pharmacological properties. Kang et al. [23] found puerarin's potential antitumor effect in non-small-cell lung carcinoma xenograft model. Other studies [24, 25] proved that puerarin might be beneficial to lipid metabolism and prevent lifestyle-related diseases. Li et al. [26] and Zhong et al. [27] showed hard evidence for puerarin's beneficial effect in diabetic animals. In our previous studies [13, 15], puerarin was found to retard cardiac hypertrophy and apoptosis in hearts of mice subjected to TAC and also puerarin could attenuate inflammatory response and apoptosis in LPS-stimulated cardiomyocytes. As for fibrosis, puerarin protected against chemical-induced hepatic fibrosis in rodents [28, 29]. Particularly, in cardiac fibrosis, puerarin was capable of inhibiting this pathological process in mice with myocardial infarction [30]. However, what puerarin does in pressure overload-induced cardiac fibrosis is not fully elucidated.

EndMT is one form of EMT and is well known for its role in the atrioventricular cushion formation during embryonic heart development [31]. In recent studies, EMT is believed to play a part in many diseases' occurrence and progress, such as idiopathic portal hypertension [32], pulmonary arterial hypertension [33], atherosclerosis [34], tumor metastasis, and the fibrotic lesions of some vital organs [35]. By performing lineage analysis of fibroblasts recruited to myocardium in Tie1Cre;R26RstoplacZ double-transgenic and FSP1-GFP transgenic mice subjected to aortic banding, Zeisberg and the colleagues [16] found EndMT contributing to cardiac fibrosis. The same results were verified again in diabetes mellitus-induced cardiac fibrosis model, in which Widyantoro et al. [36] found that EndMT was responsible for the emergence of fibroblasts from endothelial cells and about 15–20% of fibroblasts coexpressed both CD31 and FSP1, compared to 27–35% proportion in Zeisberg et al.'s research [16]. Consistent with these studies, although using an indirect way, we found HUVECs underwent phenotypic and biological behavior transition after TGF-*β*1 treatment and were attributed to at least a part of the fibrogenesis. And with puerarin pretreatment, cardiac fibrosis and EndMT were effectively blunted. These results pave the way for puerarin's prospect in treating fibrosis diseases.

PPAR-*γ* is a nuclear hormone receptor and is known for its pleiotropic roles in regulating various genes participating in lipid metabolism, glucose homeostasis, cell differentiation, survival, and proliferation [37], as well as inflammatory responses [38, 39] and anticancer effect [40, 41]. Recent studies have revealed another important function of PPAR-*γ* as a negative regulator of fibrosis in heart [42], pulmonary hypertension [43–45], and patients with systemic sclerosis [46, 47]. PPAR-*γ* interfered with Smad-dependent promoter activity and inhibited TGF-*β*-induced collagen expression and myofibroblast transdifferentiation in normal fibroblasts [48]. The activation of PPAR-*γ* by exogenous ligand or transient expression of ectopic PPAR-*γ* could significantly mitigate TGF-*β*-induced profibrotic response [49]. Interestingly, in our study, in addition to eased EndMT and fibrogenesis, PPAR-*γ* protein expression was upregulated in mice and HUVECs treated with puerarin. This phenomenon prompted a postulation: puerarin could function as a potential agonist of PPAR-*γ* or somehow PPAR-*γ* served as the performer responsible for puerarin's inhibition effect on EndMT. This postulation was made based on some hard evidence showing PPAR-*γ* was involved with EMT: through antagonizing EMT, PPAR-*γ* activation inhibited the metastasis of two kinds of cancer cells [50]. In alveolar epithelial cells, activation of PPAR-*γ* was beneficial to mitigating TGF-*β*1-induced EMT [22].

To verify this theory, we used exogenous PPAR-*γ* agonist, pioglitazone, a kind of thiazolidinediones (TZDs) which are known for treating type 2 diabetes mellitus. As evidenced by Figure 7, pioglitazone repeated the inhibition effects on TGF-*β*1-induced EndMT and fibrosis in HUVECs, like puerarin did. The suppression level on the increase of vimentin and the decrease of CD31 were not statistically different between TGF-*β*1 + Pue and TGF-*β*1 + Pio group. To explore whether this effect was PPAR-*γ*-dependent, we used the specific antagonist of PPAR-*γ*, GW9662. In the same figure, the suppression effects imposed by puerarin were sabotaged by GW9662, as shown by the statistically different changes of CD31 protein and other profibrotic genes (Fn, CTGF, and *α*-SMA) between TGF-*β*1 + Pue and TGF-*β*1 + Pue + GW9662 group. These results indicated that puerarin likely exerted its protective effect through the upregulation of PPAR-*γ*.

TGF-*β*1/Smads signaling pathway is one of the most classical pathways underlying fibrogenesis and EMT process [51–55]. Any target aiming to cut off TGF-*β*1/Smads is promising to be antifibrogenic reagent. In our study, puerarin could blunt Smad2 phosphorylation activation in a dose-dependent way but pioglitazone could not (Figure 7(a)). That is because, instead of abrogating the phosphorylation and nuclear translocation of Smad2/3, PPAR-*γ* targeted the transcriptional coactivator and histone acetyltransferase p300 in nucleus and interfered with Smad2/3 binding to the promoters of profibrotic genes [49]. That probably explained the unchanged protein level of p-Smad2 in HUVECs treated with pioglitazone.

There are some limitations in our study. In addition to Smads, c-Jun NH_2_-terminal kinase and mitogen-activated protein kinases (MAPKs) are contributing to noncanonical TGF-*β* signaling pathway [56]. We did not test their roles in this article. This calls for more profound study.

In conclusion, our study indicated that puerarin protects against cardiac fibrosis induced by pressure overload, and this protective effect may be exerted by upregulation of PPAR-*γ* and inhibiting TGF-*β*1/Smad2-induced EndMT. Puerarin may provide a new adjuvant for antifibrotic treatment strategies for patients with heart disease.

## Figures and Tables

**Table 1:** Gene-specific primers used in quantitative real-time PCR.

Gene	Forward	Reserve	Gene accession number
GAPDH	5′-CATGAGAAGTATGACAACAGCCT-3′	5′-AGTCCTTCCACGATACCAAAGT-3′	NM_001256799.2
CD31	5′-GAGTCCTGCTGACCCTTCTG-3′	5′-TCAGGTTCTTCCCATTTTGC-3′	NM_000442.4
Vimentin	5′-GAAATTGCAGGAGGAGATGC-3′	5′-ATTCCACTTTGCGTTCAAGG-3′;	NM_003380.3
*α*-SMA	5′-CTGAGCGTGGCTATTCCTTC-3′	5′-AGAAGAGGAAGCAGCAGTGG-3′	NM_001141945.2
Collagen I	5′-ACCTGGTCAAACTGGTCCTG-3′	5′-CCTGTGGTCCAACAACTCCT-3′	NM_000088.3
Collagen III	5′-GATCAGGCCAGTGGAAATGT-3′	5′-GTGTGTTTCGTGCAACCATC-3′	NM_000090.3
CTGF	5′-GTTCCAAGACCTGTGGGATG-3′	5′-TCTCTTCCAGGTCAGCTTCG-3′	NM_001901.2
Fn	5′-CAAACTCCGTCACCCTCAGT-3′	5′-GGTGCCAGTGGTTTCTTGTT-3′	NM_001306129.1

CTGF: connective tissue growth factor; Fn: fibronectin.
